# Is evaluation of cortisol after dexamethasone suppression test enough? Analysis of steroid profile after dexamethasone suppression test using tandem mass spectrometry

**DOI:** 10.1530/EC-25-0281

**Published:** 2025-09-04

**Authors:** Tomas Brutvan, Marcela Kotasova, Adela Krausova, Jarmila Krizova, Otakar Psenicka, Jan Sevcik, Martin Sevcik, Hana Vitkova, Jana Jezkova

**Affiliations:** ^1^ 3rd Department of Internal Medicine, General University Hospital in Prague and The 1st Faculty of Medicine of Charles University, Prague, Czech Republic; ^2^ Institute of Clinical Biochemistry and Laboratory Medicine, General University Hospital in Prague and The 1st Faculty of Medicine of Charles University, Prague, Czech Republic; ^3^ Transplantation Centre, Institute of Clinical and Experimental Medicine, Prague, Czech Republic; ^4^ Department of Internal Medicine of Military University Hospital and The 1st Faculty of Medicine of Charles University, Prague, Czech Republic

**Keywords:** Cushing’s syndrome, dexamethasone suppression test, tandem mass spectrometry, adrenal incidentaloma, steroid profile

## Abstract

**Introduction:**

The overnight dexamethasone suppression test (DST) is recommended for the initial testing of Cushing’s syndrome. Simultaneous measurement of dexamethasone and cortisol is recommended. This study aimed to determine the cutoff value for dexamethasone measured in DST analyzed via liquid chromatography tandem mass spectrometry (2D-LC-MS/MS) and to assess whether analysis of adrenal steroids other than cortisol may improve diagnostic accuracy.

**Methods:**

A prospective study was conducted including patients with adrenal incidentalomas (*n* = 55), pituitary incidentalomas and symptoms of Cushing’s disease (*n* = 18), and healthy controls (*n* = 100) undergoing DST. Plasma levels of ten steroids and dexamethasone were determined via 2D-LC-MS/MS, while cortisol levels were also determined via a chemiluminescence immunoassay.

**Results:**

The lower 2.5th percentile of plasma dexamethasone in the control group (cortisol level <50 nmol/L) was 3.48 nmol/L, which was set as the cutoff. In the subgroup of patients with adrenal incidentalomas, regardless of the results of DST, subjects exhibited identical changes in the basal adrenal steroid profile: increased 11-deoxycortisol and decreased DHEA and DHEAS levels. After 1 mg dexamethasone administration, cortisol and cortisone levels increased, while decreased androstenedione and 17-OHP levels were detected. Statistically significant changes were found in the subgroup of patients with pituitary incidentalomas: increased levels of 11-deoxycortisol, plasma cortisol, cortisone, and androstenedione were observed only in those with serum cortisol levels >50 nmol/L. Based on the ROC curves, none of the steroid hormones exhibited higher specificity than cortisol.

**Conclusion:**

Simultaneous measurement of cortisol and dexamethasone increases DST specificity. The cutoff value for dexamethasone was set at 3.48 nmol/L. None of the adrenal steroids in the DST demonstrated increased specificity; thus, a cortisol concentration <50 nmol/L remains the gold standard for ruling out Cushing’s syndrome.

## Introduction

Endogenous Cushing’s syndrome (CS) is a rare yet severe endocrine disorder. This syndrome is caused by chronic exposure to excess glucocorticoids. Endogenous CS is broadly classified as adrenocorticotropic hormone (ACTH)-dependent (80–85% of cases) or ACTH-independent (15–20% of cases) Cushing’s syndrome. In most cases, ACTH-dependent CS is caused by overproduction of ACTH by a pituitary neuroendocrine tumor; rarely, it is caused by ectopic paraneoplastic ACTH secretion. ACTH-independent hypercortisolism, which is characterized by adrenal tumors or adrenal hyperplasia, is less common (1, 2). Central obesity, a rounded face, facial plethora, proximal muscle weakness, spontaneous bruising, purple striae, dyslipidemia, hypertension, impaired glucose metabolism, and osteoporosis are features associated with endogenous hypercortisolism. Untreated CS is associated with increased morbidity and mortality. Therefore, early diagnosis and prompt treatment of excess cortisol levels are necessary (3).

The dexamethasone suppression test (DST) (1 mg overnight) represents one test that is recommended for the initial testing of individuals with suspected Cushing’s syndrome. For this test, a serum cortisol concentration of 50 nmol/L (1.8 μg/dL) is a widely used cutoff value exhibiting high sensitivity (95%) and moderate specificity (80%), thus demonstrating a high negative predictive value. A cortisol cutoff value of 50 nmol/L is generally recommended, although it is well known that cortisol measurement by immunoassay can be influenced by the diagnostic kit used, changes in assay performance over time, and patient sex. Moreover, one advantage of this test is that it is easy to perform (4).

Research has demonstrated that false-positive results may be observed despite the high sensitivity of the DST. This may be due to insufficient serum concentration of dexamethasone (DXM), which may be caused by several factors, including variable gastrointestinal absorption of dexamethasone, impaired hepatic or renal function, individual variations in DXM metabolism, glucocorticoid receptor polymorphisms, drugs interfering with cytochrome P450 (CYP3A4) activity, or patient noncompliance (5, 6, 7, 8, 9). Therefore, simultaneous measurement of cortisol and dexamethasone is recommended to reduce false-positive results and improve diagnostic accuracy (4). Modern, highly sensitive, and specific liquid chromatography tandem-mass spectrometry (LC-MS/MS) is currently used for the assessment of these steroids. In addition to dexamethasone and cortisol, other steroids can be simultaneously analyzed, thus representing another advantage of the method (10, 11, 12).

For the correct interpretation of the test, knowledge of the cutoff value of DXM in the DST is essential. The DXM concentration has been previously determined via radioimmunoassays (RIA) (13). Currently, various LC-MS/MS methods are used, with each method exhibiting specific cutoff values. The reported values from previous studies range from 3.3 to 5.6 nmol/L, depending on the method that is used (12, 13, 14).

Thus, the main aim of our study was to determine the cutoff value for dexamethasone in the DST by using our simple two-dimensional liquid chromatography-mass spectrometry-based method (2D-LC-MS/MS) (15). Another aim of the study was to determine whether some of the simultaneously determined adrenal steroids may exhibit higher sensitivities and specificities compared with the routinely used cortisol steroid.

## Subjects and methods

This prospective study was performed from 01/2018 to 03/2023. The study included: i) patients with adrenal incidentalomas; and ii) patients with pituitary incidentalomas and symptoms suspicious for Cushing’s syndrome.

The study included 73 patients (49 females and 24 males), aged 24–82 years (median 59.5 years), and 100 healthy controls (61 females and 39 males), aged 19–78 years (median 40 years).

The patient group consisted of two subgroups. The first subgroup included 55 patients with adrenal incidentalomas (incidental findings of an adrenal mass detected on a computerized tomography scan that was performed for reasons other than suspected adrenal disease). Among these 55 patients, 35 exhibited unilateral adrenal expansion and 20 exhibited bilateral adrenal expansion. All patients underwent a noncontrast computed tomography (CT) scan and were clinically evaluated for Cushing’s syndrome. Based on CT scan characteristics, unilateral adenomas were identified in all 35 patients with unilateral adrenal expansion. Among 20 patients with bilateral adrenal expansion, 18 had bilateral adenomas, and 2 had bilateral macronodular adrenal hyperplasia.

The second subgroup consisted of 18 patients with pituitary incidentalomas (a lesion of the pituitary gland discovered via imaging, which was performed for reasons unrelated to pituitary disease); these patients were subsequently referred to our department for endocrinological evaluation. These patients were demonstrated to exhibit symptoms that could indicate possible Cushing’s syndrome on initial clinical examination (Table 1). Thus, the patients underwent dedicated dynamic contrast-enhanced magnetic resonance imaging (MRI) of the sellar region.

**Table 1:** Symptoms and signs suggestive of Cushing’s syndrome in patients with pituitary incidentaloma with their occurrence.

Symptoms and signs	Occurrence
Central obesity, weight gain	16
Fatigue	8
Arterial hypertension	7
Dyslipidemia	5
Impaired glucose metabolism	2
Menstrual irregularities	8
Psychiatric disorders	2
Moon face	5
Facial plethora	3
Buffalo hump	2
Purple striae	3
Spontaneous bruising	4
Osteoporosis	2
Hirsutism	1
Erectile dysfunction	1
Proximal muscle weakness	2

None of the patients were taking medications known to influence dexamethasone metabolism.

The control group consisted of 100 healthy volunteers recruited from the hospital staff as well as their family members. The exclusion criteria for participation in the study included the use of oral contraceptives, hormone replacement therapy, corticosteroids, and medications known to disrupt dexamethasone metabolism by influencing cytochrome P-450 3A4 activity (Table 2). These medications had to be discontinued for at least 3 months before the study for eligibility. All subjects in the control group did not present with any signs of Cushing’s syndrome.

**Table 2:** Medication interfering with cytochrome P-450 3A4 metabolism.

Inhibition	Induction
Fluoxetine	Phenytoin
Diltiazem	Carbamazepine
Cimetidine	Nifedipine
Ritonavir	Topiramate
Itraconazole	Rifapentine

The hospital ethics committee approved the study protocol, and the subjects signed informed consent forms.

Body mass index (BMI) was calculated and recorded for the patients in this study. Fasting blood samples for basic biochemistry and adrenal steroid measurements were obtained from the subjects. Creatinine levels were analyzed using an enzymatic assay (Roche Diagnostic, Germany).

Based on the Endocrine Society Clinical Practice Guidelines for Cushing’s syndrome, the 1 mg overnight DST was used as the initial screening test. Dexamethasone (1 mg) was orally administered at 23:00 h. Blood was collected the next morning between 08:00 and 08:30 h for adrenal steroid and dexamethasone measurements.

Subjects who failed to achieve post-dexamethasone serum cortisol suppression to less than 50 nmol/L underwent further laboratory evaluations to confirm or exclude Cushing’s syndrome. Additional laboratory evaluations included ACTH, urinary free cortisol, and midnight serum cortisol measurements. The exclusion criteria for overt cortisol secretion in subjects without sufficient suppression in the DST included an awake late-night serum cortisol level ≤200 nmol/L, a urinary free cortisol level (performed two times) in the normal range, and an unsuppressed ACTH level in patients with adrenal expansions. Repeat endocrinological testing was performed, and each patient’s clinical status was observed for 2 years to definitively exclude hypercortisolism.

For the laboratory assessments, the serum cortisol level was determined by a chemiluminescence immunoassay-CLIA (Siemens Atellica, Germany); moreover, the plasma adrenocorticotropic hormone (ACTH) level was determined via an immunoradiometric assay (Lacomed, Czech Republic), and the urinary free cortisol level was determined via a radioimmunoassay (Beckman Coulter, USA). The levels of plasma steroids (including corticosterone, 11-deoxycorticosterone, 11-deoxycortisol, 21-deoxycortisol, cortisol, cortisone, 17-alpha-hydroxyprogesterone, androstenedione, dehydroepiandrosterone, and dehydroepiandrosterone sulfate) and dexamethasone (DXM) were determined by liquid chromatography with tandem mass spectrometry (2D-LC-MS/MS) (using internal standards MIX MassChrom® Panel 1.2; Chromsystems Instruments & Chemicals GmbH; Dexamethasone solution, Cerilliant®, USA). All samples were appropriately processed immediately after collection and subjected to real-time analysis.

### 2D-LC-MS/MS

An in-house method based on 2D-LC-MS/MS was used for the multiplex analysis of steroid hormones and dexamethasone in a plasma sample. This method enables direct analysis of a plasma sample after a simple process of protein precipitation followed by phase partitioning. The analysis consisted of sample preparations (each occurring for 10 min), followed by an 18-min analytical run.

For the chemicals, dexamethasone solution (DXM; 1.0 mg/mL in methanol) purchased from Sigma-Aldrich at the certified reference material grade was used. The internal standard dexamethasone-D4 (DXM-D4) was obtained from Cayman Chemical Company. The other utilized chemicals were the same as those used in a previous study (15). A stock solution of DXM at a concentration of 10.19 μmol/L and DXM-D4 (10.09 μmol/L) was prepared in LC-MS grade methanol. The dexamethasone calibration curve and quality controls (QCs) were prepared by diluting the stock solution and mixing it in pooled plasma. The final concentrations were in the range of 1.83–58.48 nmol/L for calibration and 2.09, 8.35, and 33.42 nmol/L for QC. The concentration of DXM-D4 in the ACN solution was 10 nmol/L. All of the solutions were stored at −25°C for a maximum of 3 months. The preparation of the samples and the chromatographic settings were the same as those described in a previously published method (15). In contrast, a mixture of DXM-D4 and the internal standard known as MIX MassChrom® Steroids was added to the serum/plasma. Therefore, simultaneously measuring dexamethasone and ten steroid hormones in one run was possible. The mass transitions, retention times, and cell voltages were 393.2→373.2 *m*/*z*, 10.8 min, and 4 V, respectively, for dexamethasone, and 397.2→377.2 *m*/*z*, 10.7 min, and 8 V, respectively, for dexamethasone-D4. Moreover, the fragmentor voltage was set to 108 V.

Precision and accuracy were performed in ten replicates on all levels of the QC samples traceable to the reference material. The precision results revealed that the relative standard deviation (RSD) of all QC levels ranged from 0.81 to 1.5% within the runs and 2.2–2.3% between the runs. The accuracy varied between 98.2 and 104.2%.

Recovery was determined by spiking low and high QCs into pooled plasma samples in five repetitions before and after extraction. The matrix effect (ME) was calculated by comparing the peak area of the analyte based on the spiking of the different concentrations into the postextraction matrix of six different samples with the peak area based on the spiking into the solvent matrix. The mean recovery was 122% (RSD: 3.4%) for the absolute samples and 93.5% (RSD: 3.8%) for the relative samples. The MEs were in the range of 96.7–98.0% (RSD: 2.5–2.7%). The method detection limit was again used as a parameter of sensitivity. The obtained value was 0.09 nmol/L.

Calibration curves were constructed for different concentration ranges by diluting the stock solution of DXM in pooled plasma. Linear regression was used to construct calibration curves (*y* = 0.107x-0.020; RSD = 0.5%), with squared regression coefficients of R2 = 0.9999 and a weight of 1/x.

### Statistical analysis

Statistical analyses were performed using R-commander version 4.4.1 (GNU General Public License) and MATLAB R2023B (The MathWorks). The subject characteristics are reported as medians (ranges). The dexamethasone cutoff for a sufficient DST was calculated as the lower 2.5th percentile of individuals in the control group, with suppression of serum cortisol below 50 nmol/L. The Wilcoxon rank-sum test was used to compare steroid profiles between the groups. Spearman correlation was used to determine the associations between DXM levels and creatinine levels, age, and BMI. To establish a steroid hormone cutoff for the DST, we used receiver operating characteristic (ROC) analysis in the control group patients with cortisol levels less than 50 nmol/L (CLIA) and DXM levels above 3.48 nmol/L (LC-MS/MS). In addition, we established thresholds with respective sensitivity and specificity only for steroid hormones with areas under the curve (AUCs) greater than 0.7. Statistical significance was set at *P* < 0.05.

## Results

### Dexamethasone cutoff and DST results

The median DXM concentration after the DST in all of the cohorts was 9.58 nmol/L (range: 1.85–24 nmol/L); moreover, the concentration in the control group was 8.82 nmol/L (range: 2.6–24 nmol/L), and the concentration in the patient group was 10.95 nmol/L (range: 1.85–20.4 nmol/L). The median value of serum cortisol analyzed via CLIA after the DST in all of the cohorts was 29.35 nmol/L (range: 14.3–1,170 nmol/L); additionally, the median value in the control group was 24.2 nmol/L (range: 14.3–157 nmol/L), and the value in the patient group was 43.55 nmol/L (range: 19–1,170 nmol/L). All these data and detailed cohort characteristics are presented in Table 3. A cortisol level of less than 50 nmol/L was achieved in 90 (90%) control group subjects. These subjects were used to determine the cutoff value for DXM. The lower 2.5th percentile of DXM was 3.48 nmol/L (3.5 nmol/L), which was established as the cutoff value for the DST with 1 mg dexamethasone. This cutoff value was achieved in 95 (95%) subjects in the control group. We observed no correlation between creatinine levels (which reflect renal function) and plasma dexamethasone levels, nor between sex and plasma dexamethasone levels.

**Table 3:** Cohort characteristics and results of DST (1 mg).

	All cohort median (range)	Patient median (range)	Control group median (range)
*n*	174	74	100
Sex (F/M)	110/63	49/24	61/39
Age	48 (19–82)	59.5 (24–82)	40 (19–78)
BMI (kg/m^2^)	28 (15–71.4)	29 (15–50)	27 (17.5–71.4)
Creatinine (μmol/L)	72 (41–249)	69 (41–249)	73.5 (46–203)
Cortisol in DST (1 mg) CLIA (nmol/L)	29.35 (14.3–1,170)	43.5 (18–1,170)	24.2 (14.3–157)
Cortisol in DST (1 mg) 2D-LC-MS/MS (nmol/L)	29.3 (12–852)	46.53 (18–852)	24.9 (12–160)
Dexamethasone 2D-LC-MS/MS (nmol/L)	9.8 (1.85–24)	10.95 (1.85–20.4)	8.82 (2.6–24)

Age was positively correlated with DXM in the whole cohort and in the control group, but in both with low correlation coefficient values (*r* = 0.24 and *r* = 0.19, respectively). BMI was positively correlated with DXM in the whole cohort, as well as in the patient and control groups (Table 4).

**Table 4:** Correlation coefficients between dexamethasone and creatinine, age, and BMI.

		*P* value	*R*
Creatinine	All cohort (*n* = 174)	0.54	0.05
	Patients (*n* = 74)	0.3	0.12
	Controls (*n* = 100)	0.91	0.01
Age	All cohort (*n* = 174)	**<0.01**	0.24
	Patients (*n* = 74)	0.45	0.09
	Controls (*n* = 100)	0.05	0.19
BMI	All cohort (*n* = 174)	**<0.01**	0.29
	Patients (*n* = 74)	**0.02**	0.26
	Controls (*n* = 100)	**<0.01**	0.28

Bold indicates statistical significance (*P* < 0.05).

In the patient group, suppression of serum cortisol in the DST <50 nmol/L (CLIA) was achieved in 39 patients (53%). All of these patients demonstrated sufficient DXM (DXM >3.5 nmol/L). In 34 patients (47%), the serum cortisol level was >50 nmol/L (CLIA). Moreover, all but one patient obtained sufficient DXM. In this group of 34 patients, further endocrinological examinations ruled out hypercortisolism in 16 patients, including one patient with an insufficient DXM level. Overt Cushing’s syndrome was confirmed in 11 patients. Furthermore, seven patients were observed to have possible autonomous cortisol secretion; therefore, they required regular endocrinological follow-up examinations.

In the control group, sufficient suppression of serum cortisol was achieved in ninety (90%) subjects. Two subjects from the control group did not achieve a sufficient DXM level; however, their serum cortisol level was <50 nmol/L (CLIA) on the DST. Ten individuals (10%) had a serum cortisol concentration >50 nmol/L (CLIA), three of whom did not obtain the DXM cutoff level. In all ten individuals, hypercortisolism was excluded based on further endocrinological examinations.

### Steroid profile

All of the samples from the patients and the control group were analyzed via 2D-LC-MS/MS for the steroid hormone spectrum and DXM. We observed a statistically significant difference between the cortisol levels analyzed via CLIA and 2D-LC-MS/MS (*P* < 0.01) in both groups. The 2D-LC-MS/MS basal morning cortisol level was 3.27% lower than the cortisol level measured by immunoassay.

Basal morning plasma cortisol concentrations were statistically significantly higher in the patient group than in the control group, as were the basal 11-deoxycortisol and 11-deoxycorticosterone concentrations. In contrast, the basal levels of dehydroepiandrosterone (DHEA) and dehydroepiandrosterone sulfate (DHEAS) were statistically significantly lower. Basal levels of androstenedione, 17-α-hydroxyprogesterone (17-OHP), corticosterone, 21-deoxycortisol, and cortisone were without statistical difference between these two groups.

When basal steroid hormone levels in a subgroup of patients with adrenal expansion and serum cortisol levels >50 nmol/L (CLIA) on the DST were compared with those in the control group, statistically significantly higher basal levels of 11-deoxycortisol and 11-deoxycorticosterone, as well as statistically significantly lower basal levels of androstenedione, DHEA, and DHEAS, were observed in the patient group. Patients with adrenal expansions and cortisol levels >50 nmol/L (CLIA) on the DST were further divided into two subgroups: those with mild autonomous cortisol secretion (MACS) and those with overt Cushing’s syndrome. When comparing the subgroup of patients with MACS to the control group, the same steroid changes were demonstrated as for the entire group of patients with adrenal expansions, with the exception of 11-deoxycorticosterone, where no statistically significant difference was found. Due to the limited number of patients with overt Cushing’s syndrome, statistical analysis and comparison with the control group were not possible. Patients with adrenal expansion and serum cortisol levels <50 nmol/L (CLIA) on the DST exhibited statistically significantly higher basal levels of 11-deoxycortisol and lower basal levels of DHEA and DHEAS than did the control group (Table 5).

**Table 5:** Basal steroid profile (2D-LCMS/MS): correlation between subgroups and control group.

	Cortisol	11-deoxycortisol	21-deoxycortisol	Corticosterone	Cortisone	11-deoxycorticosterone	17-alpha-hydroxyprogesterone	Androstenedione	DHEA	DHEAS
*P value*										
Patient group vs controls	**0.02**	**<0.01**	0.4	0.66	0.26	**0.02**	0.93	0.08	**<0.01**	**<0.01**
Adrenal expansions with cortisol <50 nmol/L in DST (1 mg) vs controls	0.37	**0.02**	0.31	0.61	0.31	0.11	0.88	0.23	**<0.01**	**<0.01**
Adrenal expansions with cortisol >50 nmol/L in DST (1 mg) vs controls	0.18	**<0.01**	0.66	0.13	0.49	**0.03**	0.29	**<0.01**	**<0.01**	**<0.01**
Pituitary tumor with cortisol<50 nmol/L in DST (1 mg) vs controls	0.35	0.21	0.83	0.22	0.11	0.66	0.17	0.54	0.92	0.38
Pituitary tumor with cortisol >50 nmol/L in DST (1 mg) vs controls	**<0.01**	**<0.01**	0.43	0.33	**0.01**	0.25	0.71	**0.01**	0.85	0.46

Bold indicates statistical significance (*P* < 0.05).

Compared with the control group, the subgroup of patients with pituitary incidentalomas and serum cortisol levels >50 nmol/L (CLIA) in DST exhibited statistically significantly higher basal levels of 11-deoxycortisol, plasma cortisol, cortisone, and androstenedione. There were no statistical differences observed in the basal steroid profile between the subgroup of patients with pituitary incidentalomas and those with serum cortisol levels <50 nmol/L (CLIA) in the DST and control groups.

A comparison of the results of the steroid profile after the administration of 1 mg dexamethasone via the DST revealed statistically significantly higher levels of plasma cortisol, 11-deoxycortisol, and cortisone, as well as statistically significantly lower levels of androstenedione, 17-OHP, DHEA, and DHEAS, in the patient group than in the control group.

Regarding the subgroups, after the administration of 1 mg dexamethasone, patients with adrenal expansion and serum cortisol concentrations >50 nmol/L (CLIA) exhibited statistically significantly higher levels of 11-deoxycortisol, plasma cortisol, and cortisone, as well as statistically significantly lower levels of 17-OHP, androstenedione, DHEA, and DHEAS, than did the control group. No statistically significant lower levels of 17-OHP were found in patients with adrenal incidentalomas and MACS. The rest of the steroid profile was identical to that of the whole adrenal expansion group. Moreover, patients with adrenal expansion and serum cortisol levels <50 nmol/L (CLIA) exhibited statistically significantly higher levels of 11-deoxycortisol, plasma cortisol, and cortisone, as well as significantly lower levels of 17-OHP, androstenedione, DHEA, and DHEAS.

Compared with those in the control group, statistically significantly higher levels of 11-deoxycortisol, plasma cortisol, cortisone, and androstenedione were detected after the administration of 1 mg dexamethasone in the subgroup of patients with pituitary incidentalomas and serum cortisol levels >50 nmol/L (CLIA). In contrast, there were no statistical differences observed between the subgroup of patients with pituitary incidentalomas and those with serum cortisol concentrations <50 nmol/L (CLIA) after the administration of 1 mg dexamethasone compared with the control group (Table 6).

**Table 6:** Steroid profile in DST (1 mg) (2D-LC-MS/MS): correlation between subgroups and control group.

	Cortisol	11-deoxycortisol	21-deoxycortisol	Corticosterone	Cortisone	11-deoxycorticosterone	17-alfa-hydroxyprogesterone	Androstenedione	DHEA	DHEAS
*P value*										
Patient group vs controls	**<0.01**	**<0.01**	0.62	0.2	**<0.01**	0.99	**0.01**	**<0.01**	**<0.01**	**<0.01**
Adrenal expansions with cortisol <50 nmol/L in DST (1 mg) vs controls	**<0.01**	**<0.01**	0.8	0.13	**<0.01**	0.28	**<0.01**	**<0.01**	**<0.01**	**<0.01**
Adrenal expansions with cortisol >50 nmol/L in DST (1 mg) vs controls	**<0.01**	**<0.01**	0.18	**<0.01**	**<0.01**	0.23	0.05	**<0.01**	**<0.01**	**<0.01**
Pituitary tumor with cortisol <50 nmol/L in DST (1 mg) vs controls	0.63	0.24	0.23	0.82	0.86	0.8	0.94	0.94	0.63	0.85
Pituitary tumor >50 nmol/L in DST (1 mg) vs controls	**<0.01**	**<0.01**	**0.03**	0.35	**<0.01**	0.61	0.87	**0.02**	0.15	0.31

Bold indicates statistical significance (*P* < 0.05).

### Steroid threshold

**Figure 1:**
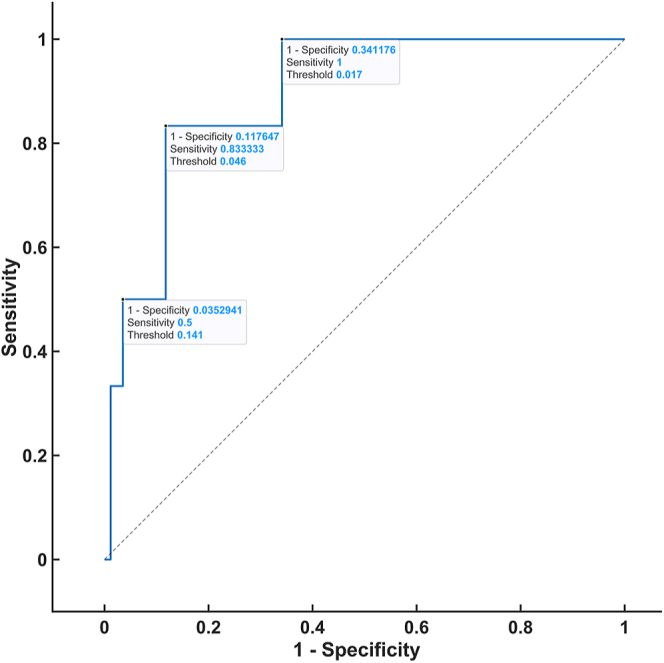
ROC curve of 21-deoxycortisol in the DST.

**Figure 2:**
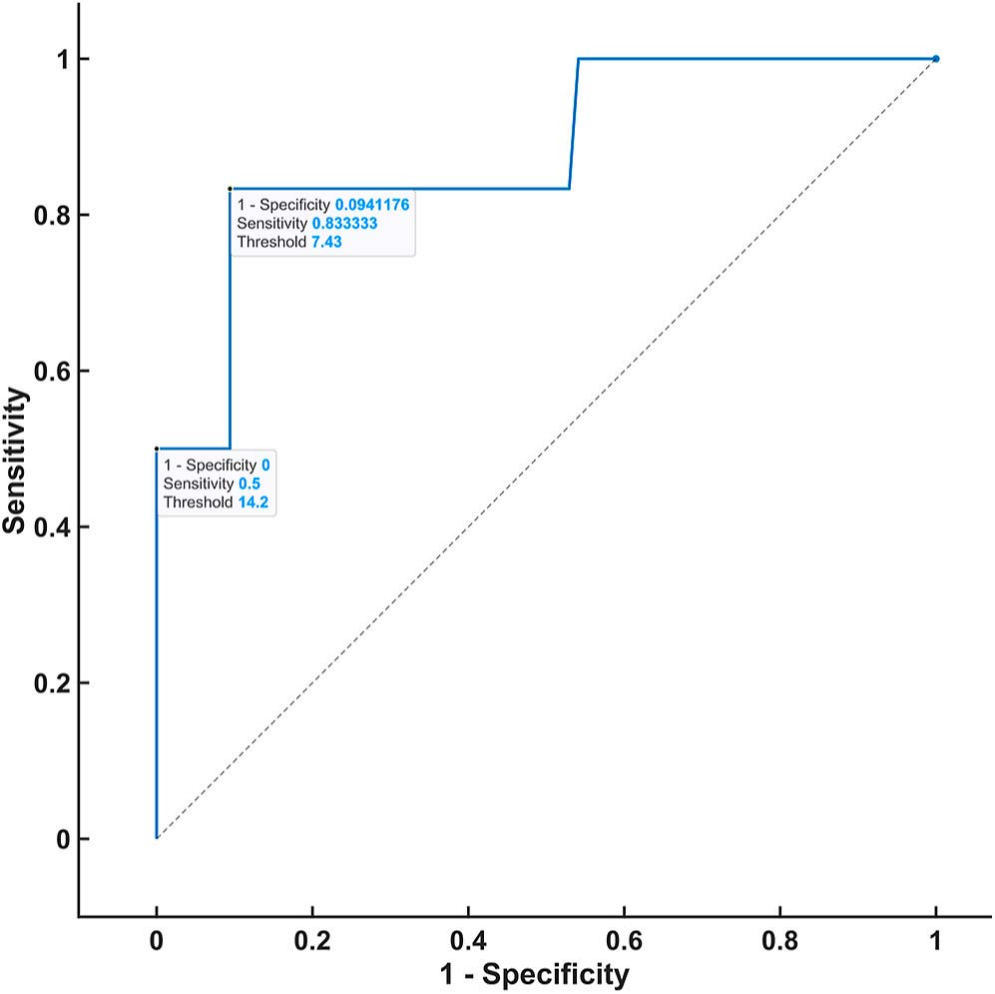
ROC curve of cortisone in the DST.

**Figure 3:**
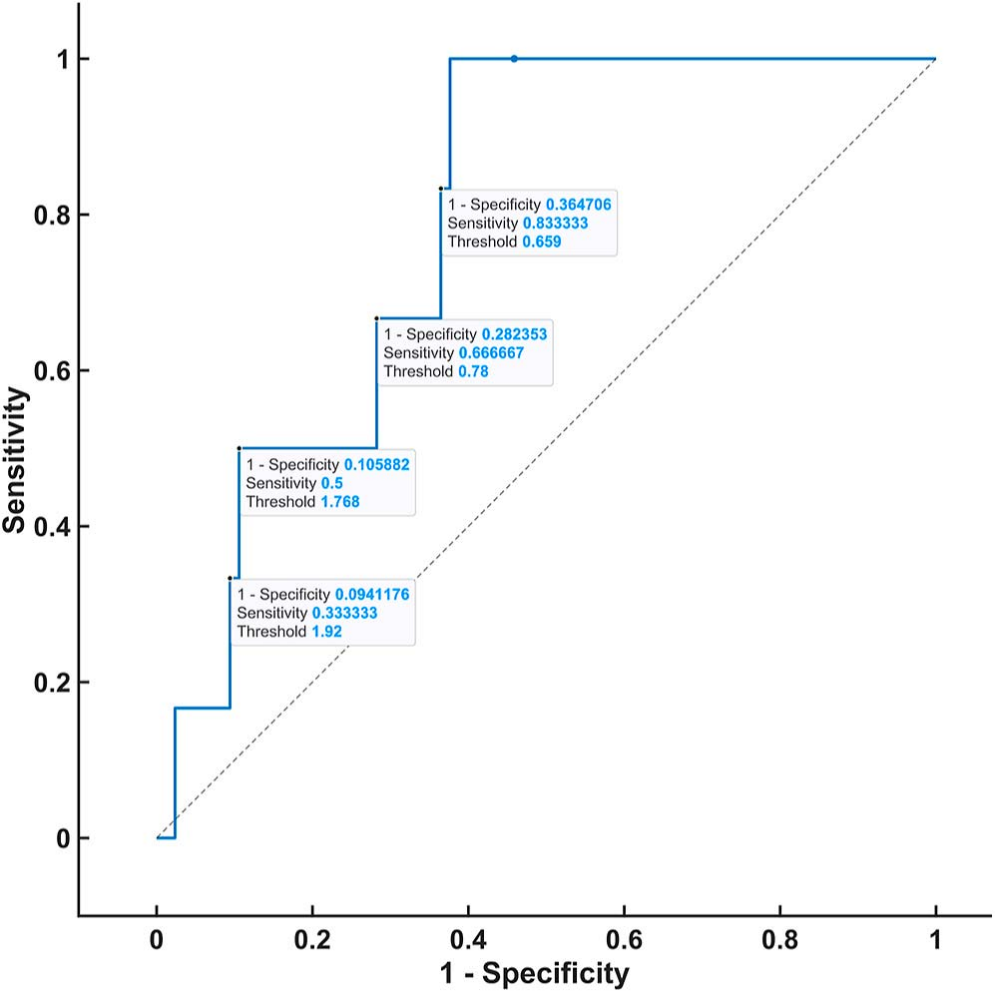
ROC curve of corticosterone in the DST.

**Figure 4:**
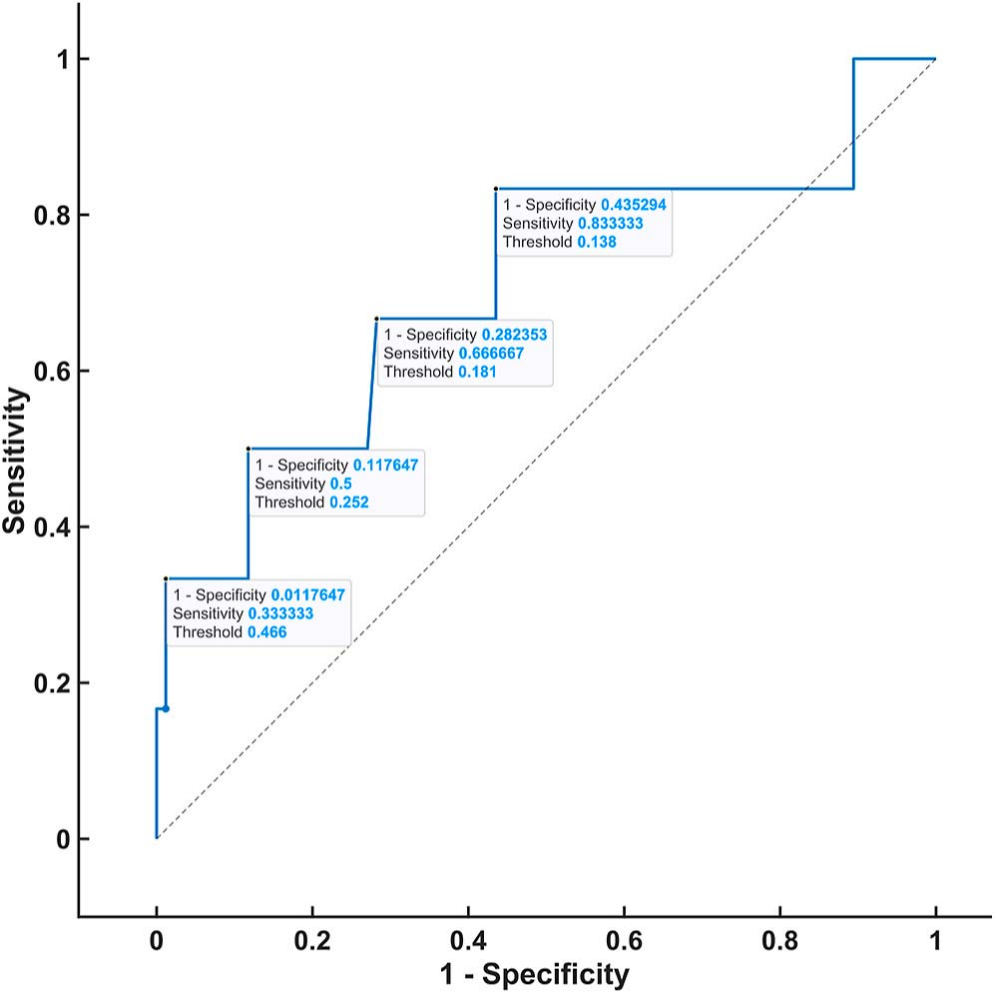
ROC curve of 11-deoxycortisol in the DST.

To establish the threshold of steroid hormones in the DST that were analyzed via 2D-LC-MS/MS, ROC analysis was performed by constructing ROC curves and obtaining thresholds with respective true positive rates (sensitivity) and false-positive rates (1-specificity). The ROC analysis was based on patients in the control group who fulfilled both of the following conditions: 1) plasma dexamethasone level ≥3.5 nmol/L and 2) serum cortisol level <50 nmol/L (CLIA) on the DST. The ROC curve was plotted for all of the steroids for which a statistically significant difference was proven after the administration of 1 mg dexamethasone between the patients and controls. The highest AUC values were observed for 21-deoxycortisol (0.8941), cortisone (0.8794), corticosterone (0.7922), and 11-deoxycortisol (0.7108) (Figs 1, 2, 3, 4). For the remaining steroids, the AUC value was less than 0.51. The thresholds with calculated sensitivity and specificity values are found in Table 7. The plasma cortisol threshold that was analyzed via 2D-LC-MS/MS was established as 47.5 nmol/L, with a sensitivity of 100% and a specificity of 98%.

**Table 7:** Steroid threshold based on 2D-LC-MS/MS.

	Threshold (nmol/L)	Sensitivity (%)	Specificity (%)
Cortisol (AUC 0.9975)	47.5	100	98
	54	86	100
21-deoxycortisol (AUC 0.8941)	0.017	100	66
	0.046	84	88
	0.141	50	93
Cortisone (AUC 0.8794)	7.43	84	91
	14.2	50	100
Corticosterone (AUC 0.7922)	0.659	84	64
	0.78	67	72
	1.768	50	89
	1.92	34	91
11-deoxycortisol (AUC 0.7108)	0.138	84	56
	0.181	67	72
	0.252	50	88
	0.466	34	99

## Discussion

DST (1 mg) is characterized by high sensitivity (95%) and moderate specificity (80%). The simultaneous measurement of dexamethasone and cortisol is recommended to reduce false positivity and consequently increase the specificity of the DST (12, 13, 14). The determination of the method-specific dexamethasone cutoff value is essential for the accurate assessment of the DST and represented a primary objective of our study. For our in-house 2D-LC-MS/MS method, a dexamethasone concentration of 3.5 nmol/L was verified as the minimal concentration required for adequate cortisol suppression in the DST. The RIA method that has been previously used resulted in higher cutoff values for dexamethasone (12). When determined via modern and analytically more accurate LC-MS/MS methods, the cutoff levels are lower, which is consistent with the results of our study. All of the laboratories using this technique must determine their specific cutoff values before the routine use of this method (14).

We detected an insufficient DXM level (dexamethasone <3.5 nmol/L) in five subjects from the control group, representing 5% of the subjects. Nevertheless, two of these five patients achieved adequate cortisol suppression (<50 nmol/L via CLIA) after the administration of 1 mg dexamethasone. This phenomenon has been reported in other studies and may be explained by the different sensitivities of hypothalamic CRH neurons. In studies focusing on the measurement of dexamethasone during the 1 mg overnight DST, the proportion of patients who failed to exhibit suppression of serum cortisol below 50 nmol/L (CLIA) and who demonstrated a dexamethasone level below the cutoff value of the utilized method ranged from 3 to 5% (10, 12). We detected false-positive DST results due to DXM being below our cutoff value in 3% of the patients; thus, our study aligns with the findings from previous studies. Unless the individual is identified as taking a medication that interferes with CYP3A4 activity, or has a disease that leads to intestinal malabsorption, it is not possible to clearly identify other causes of inadequate dexamethasone levels (16, 17, 18).

We observed no correlation between DXM and sex; therefore, the DXM cutoff in the DST can be used without considering differences for both sexes, as demonstrated by other researchers (12, 14). In contrast to the findings of some previous studies, creatinine levels were not correlated with DXM (12). This result could be influenced by the fact that our cohort did not include a sufficient number of patients with significant renal insufficiency.

Our study revealed a positive correlation between age and dexamethasone concentration (but with a low correlation coefficient value). Other studies (including Vogg *et al.* and Ceccato *et al.*) did not identify this correlation (14, 19). Age-specific cutoff values for dexamethasone concentrations cannot be determined based on these results, but should be the aim of further studies.

**Figure 5:**
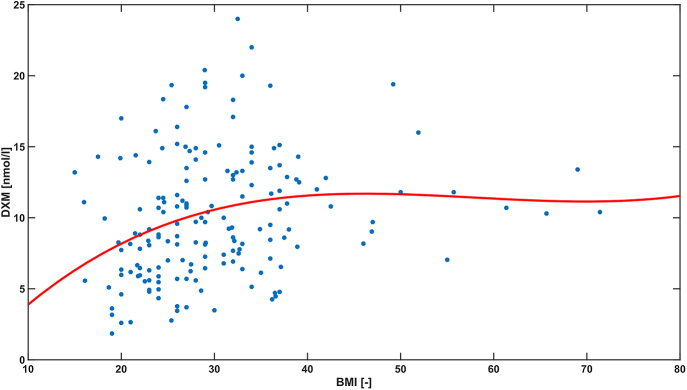
Correlation between the plasma dexamethasone concentration and BMI.

An increased number of false-positive results after the administration of 1 mg dexamethasone via the DST in obese individuals has been reported in previous studies; therefore, a 2 mg DST has been recommended as a more accurate test for this group (20). Recent studies have not confirmed these findings (21, 22). In contrast, several studies (including our study) have demonstrated a significant positive correlation between BMI and dexamethasone concentration (Fig. 5). This discrepancy may be partly explained by the assay method that is used, such as the previous determination of cortisol by a first-generation immunoassay exhibiting low assay specificity. In addition, several studies have demonstrated a negative correlation between BMI and cortisol concentrations after the DST. Ceccato *et al.* proposed several hypotheses to explain these results, such as the distribution of lipophilic DXM in the body tissues of obese patients in relation to a prolonged half-life, and greater HPA axis suppression, or increased central glucocorticoid sensitivity. An increased lag time of dexamethasone has also been observed in previous studies. Ueland *et al.* discussed the role of lower cortisol binding globulin levels in obese patients (12, 19, 21, 22, 23). Based on our results, the 1 mg DST is an appropriate screening test for obese patients with suspected Cushing’s syndrome; therefore, the use of the 2 mg DST as a screening test for obese individuals is not warranted.

In routine practice, immunoassays are most commonly used to measure cortisol (total cortisol); however, this method of analysis is burdened by a certain degree of cross-reactivity to other endogenous and exogenous steroids. The currently used second-generation immunoassays utilize monoclonal antibodies; therefore, these methods are characterized by increased specificity and reduced cross-reactivity compared with first-generation immunoassays. However, certain limitations persist, particularly related to sex-specific issues, cross-reactivity with exogenous steroids, and endogenous steroids, which are becoming more pronounced in cases of steroid hormone overexpression. In contrast, liquid chromatography-tandem mass spectrometry is the most accurate method for the determination of cortisol and other steroid hormones. LC-MS/MS is known for its high specificity due to the exclusion of interference from other endogenous or exogenous steroids. Another advantage involves the simultaneous multiplex analysis of steroids within a given sample (1, 4, 24). Importantly, the difference between the cortisol concentrations measured by using both CLIA and 2D-LC-MS/MS in our laboratory was only 3.27%. In our study, the cutoff value for cortisol in the DST (as determined via our in-house 2D-LC-MS/MS method) was 47.5 nmol/L. This value was only approximately 1% lower than the cutoff value for cortisol obtained from the DST established via CLIA. Therefore, a cortisol value of 50 nmol/L in the DST is a reliable cutoff value, even when it is determined via 2D-LC-MS/MS.

A further aim of our study was to determine whether a parameter other than cortisol can improve the accuracy of the DST. A ROC curve was constructed for each of the ten steroid hormones that were analyzed via our in-house 2D-LC-MS/MS method, which revealed a significant difference in the DST between the patients and controls. Based on the evaluation of the ROC curves of these hormones, no steroid hormone was demonstrated to have a higher specificity than the routinely used cortisol hormone.

Our results are in agreement with those of other studies focusing on the basal steroid profile of patients with adrenal expansions and insufficient cortisol suppression (cortisol >50 nmol/L via CLIA) via the DST, including higher levels of 11-deoxycortisol and 11-deoxycorticosterone, and lower levels of DHEA, DHEAS, and androstenedione compared to the control group. Increased adrenal glucocorticoid precursors are a typical finding of hyperactive steroidogenesis in patients with Cushing’s disease, whereas decreased secretion of DHEA and DHEAS in the zona reticularis is caused by low levels of ACTH in the adrenal CS (25, 26, 27). In patients with insufficient cortisol suppression in the DST, the steroid profile after the administration of 1 mg dexamethasone exhibited increased levels of plasma cortisol, 11-deoxycortisol, and cortisone, as well as decreased levels of androstenedione, 17-OHP, DHEA, and DHEAS. A similar pattern was observed in patients with adrenal expansions and sufficient cortisol suppression in the DST (cortisol <50 nmol/L via CLIA); however, the difference was statistically significant. Dalmazi *et al.* established a link between the reduced suppression of cortisone levels after the DST, with a higher prevalence of resistant hypertension due to the greater affinity of cortisone for mineralocorticoid receptors (27). In addition, DHEA is known to affect visceral fat by reducing adipocyte proliferation and activity via the inhibition of 11beta-hydroxysteroid dehydrogenase type 1. Moreover, reduced DHEA levels have been shown to contribute to increased waist circumference, and may also contribute to increased cardiovascular risk.

In contrast, in patients with pituitary nonfunctioning tumors and sufficient suppression of cortisol with the DST, there was no significant difference observed in the steroid profile between the patient and control groups.

These findings suggest that even adrenal expansion without proven autonomous cortisol secretion may be a source of mild hormonal activity, as indicated by the results of previous studies; thus, adrenal expansion may be associated with increased cardiovascular risk for patients (28, 29).

This study has several limitations. First, the distribution of patients into subgroups according to the inclusion criteria was not completely uniform. The subgroups of patients categorized according to hormone testing results were not equal in size. A larger sample of patients, individuals of the control group, and a more even distribution of study groups would certainly increase the predictive value of the statistical analysis. The cortisol cutoff value was determined using only the direct method, i.e., based on the results of a control group of healthy individuals. A further limitation was the higher proportion of bilateral adrenal incidentalomas in the adrenal incidentaloma subgroup (36%) compared to previous studies (15–17%) (30, 31). Another limitation involves the fact that individuals in the control group did not undergo adrenal imaging (CT or MRI); however, the prevalence of adrenal incidentaloma in the population is low (between 1 and 6%) (32).

In conclusion, our study highlights that the simultaneous measurement of cortisol and plasma dexamethasone reduces false-positive results and consequently increases the specificity of the DST. This scenario always requires the determination of a dexamethasone cutoff value for the utilized analytical method; specifically, in our study (which used 2D-LC-MS/MS), this value was 3.48 nmol/L. LC-MS/MS analysis allows for the multiplex determination of adrenal steroids. The determination of other adrenal steroids in the DST was not associated with an increased specificity of the test; therefore, the cortisol cutoff value of 50 nmol/L in the DST remains the gold standard for excluding Cushing’s syndrome. Autonomous cortisol secretion of adrenal origin is associated with typical changes in the adrenal steroid profile that may be associated with increased cardiovascular risk. As similar changes in the steroid profile are observed in adrenal expansions without evidence of autonomous cortisol secretion, long-term follow-up of individuals with nonfunctioning adrenal enlargement should be considered.

## Declaration of interest

The authors declare that there is no conflict of interest that could be perceived as prejudicing the impartiality of the research reported.

## Funding

This study was funded by the Ministry of Health of the Czech Republic project MH CZ-DRO (General University Hospital in Prague-VFN00064165).

## Author contribution statement

TB and JJ conceived the study. TB, JJ, MK, and JS designed the study. TB, AK, HV, JK, OP, and JJ carried out the investigation. TB, AK, HV, JK, OP, and JJ provided resources. TB, JS, and MS performed the analysis. TB, JS, MS, and JJ interpreted the data. TB, JJ, and MK were responsible for writing the original draft. JJ helped in supervision. TB, MK, AK, JS, MS, HV, JK, OP, and JJ helped in writing review and editing. All of the authors have read and agreed to the published version of the manuscript.

## Ethics statement

The study protocol was approved by the Ethics Committee of the General University Hospital in Prague, and all of the subjects signed informed consent forms.
